# Predigested Mixture of Arachidonic and Docosahexaenoic Acids for Better Bio-Accessibility

**DOI:** 10.3390/md22050224

**Published:** 2024-05-16

**Authors:** Assamae Chabni, Blanca Pardo de Donlebún, Marina Romero, Carlos F. Torres

**Affiliations:** 1 Department of Production and Characterization of Novel Foods, Institute of Food Science Research (CIAL, CSIC-UAM), C/Nicolas Cabrera 9, Cantoblanco Campus, Autonomous University of Madrid, 28049 Madrid, Spain; assamae.chabni@uam.es (A.C.); marinaromerod@outlook.com (M.R.); 2 Department of Bioactivity and Food Analysis, Institute of Food Science Research (CIAL, CSIC-UAM), C/Nicolas Cabrera 9, Cantoblanco Campus, Autonomous University of Madrid, 28049 Madrid, Spain; blanca.pardo@csic.es

**Keywords:** arachidonic acid, bio-accessibility, docosahexaenoic acid, esterification, hydrolysis, in vitro digestion, predigested

## Abstract

A predigested product from arachidonic acid oil (ARA) and docosahexaenoic acid (DHA) oil in a 2:1 (*w*/*w*) ratio has been developed and evaluated in an in vitro digestion model. To produce this predigested lipid mixture, first, the two oils were enzymatically hydrolyzed up to 90% of free fatty acids (FFAs) were achieved. Then, these two fatty acid (FA) mixtures were mixed in a 2:1 ARA-to-DHA ratio (*w*/*w*) and enzymatically esterified with glycerol to produce a mixture of FFAs, mono-, di-, and triacylglycerides. Different glycerol ratios and temperatures were evaluated. The best results were attained at 10 °C and a glycerol-to-FA molar ratio of 3:1. The bio-accessibility of this predigested mixture was studied in an in vitro digestion model. A total of 90% of the digestion product was found in the micellar phase, which contained 30% monoacylglycerides, more than 50% FFAs, and a very small amount of triacylglycerols (3% *w*/*w*). All these data indicate an excellent bio-accessibility of this predigested mixture.

## 1. Introduction

Numerous biological activities have been attributed to long-chain polyunsaturated fatty acids (LCPUFAs) [1]. However, these fatty acids (FAs) are not always readily bioavailable [2]. This is mainly attributed to their chemical structure which makes the triglycerides (TAGs) containing them more resistant to pancreatic lipase [3,4]. It has been shown that diacylglyceride (DAG) and monoacylglyceride (MAG) molecules can increase the formation of micelles and, therefore, enhance the absorption of LCPUFAs in enterocytes [5]. Therefore, the use of predigested forms of fats is a critical requirement since it has been well established that predigested mixtures are of enormous importance to prevent injuries, diseases, and to improve the bio-accessibility and bioavailability of some FAs [2,5,6,7]. As an example, Necrotizing enterocolitis (NEC), a severe gastrointestinal pathology that affects preterm infants, related to the administration of infant formula, usually evolves into complications such as lung injuries. It has been described that a nutritional formula rich in predigested triglycerides that do not require lipase activity significantly reduces NEC at least in an animal model [8].

Predigested mixtures of fats and oils can comprise non-esterified FAs (NEFAs), acylglycerol mixtures, or mainly monoacylglycerols (MAGs). These different products require much less pancreatic lipase activity and less biliary secretion to be absorbed than regular triacylglycerols, which have a low dispersant capacity. 

In a recent clinical trial [5], it has been described that MAG oil rich in omega-3 is more bioavailable compared to other omega-3 carriers, such as ethyl ester and free fatty acids (FFAs), in healthy subjects under a low-fat diet. In another study, a predigested formula containing arachidonic acid (ARA) and docosahexaenoic acid (DHA), produced by lipase-catalyzed hydrolysis, with a NEFA content up to six times higher than original triacylglycerol oil, reduced the fecal excretion of fat and increased the plasmatic levels of these two FAs [7]. However, in this study, the composition of the acylglycerol mixture produced after hydrolysis is not mentioned.

One important issue regarding the administration of predigested mixtures is the oxidative stability of these mixtures. Several studies have indicated that FFAs have pro-oxidant activity [9,10]. The proposed mechanism is the increased decomposition rate of hydroperoxides. For this reason, the viability of predigested mixtures should pay attention to oxidation status in order to provide the optimum shelf life of these mixtures [11,12].

In a previous study, we have demonstrated a better bio-accessibility of a predigested lipid formula produced via enzymatic glycerolysis with a very low content of FFAs. In this study, a predigested mixture produced via the enzymatic esterification of an FA mixture containing ARA and DHA in a ratio of 2 to 1 has been digested in an in vitro digestion model. This new predigested formula contains a higher content of FFAs and a different distribution of lower glycerides compared to that obtained via enzymatic glycerolysis. We considered that these predigested formulas can provide an interesting approach for the administration of these bioactive FAs, especially in premature newborns. 

## 2. Results and Discussion

### 2.1. Enzymatic Hydrolysis of Microalgae and Arachidonic Acid Oils

**Figure 1:**
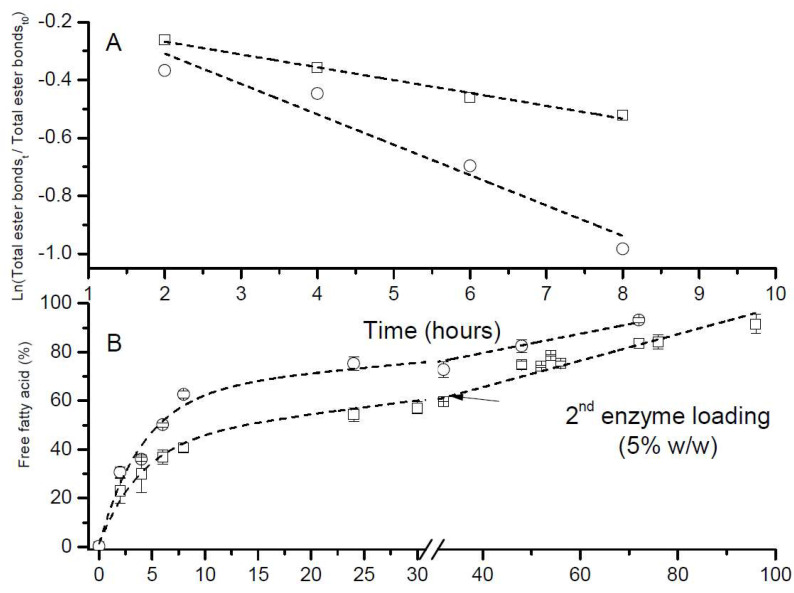
Hydrolysis rate of ARA oil 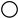 and DHA oil 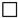 (**A**), and time course of hydrolysis reaction of ARA oil 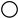 and DHA oil 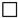 (**B**).

Initially, DHA oil and ARA oil were hydrolyzed to produce free fatty acids of both oils. The results are depicted in Figure 1. To determine the rate of each hydrolysis reaction, a linear regression of total ester bonds (%) vs. time was carried out. Assuming a first-order reaction, the linearization of this response is carried out by a semi-logarithm representation. Only the first 8 h (Figure 1A) of hydrolysis are plotted to avoid data close to the hydrolysis equilibrium. The rate of hydrolysis is obtained from the slope of each line (Table 1). As can be observed, the rate of the hydrolysis of DHA oil is much slower (slope −0.04435) than that of ARA oil (slope −0.10494) for the first 8 h of enzymatic hydrolysis. This difference in the rate of hydrolysis can be attributed to the different length chain and number of double bonds between ARA (C20:4n − 6) and DHA (C22:6n − 3) that can produce a higher discrimination of the lipase against DHA. Other authors who have carried out a similar hydrolysis of PUFA-rich anchovy oils with lipases from other sources have obtained similar results [13,14]. 

Once the rate of hydrolysis was established for both oils, the enzymatic hydrolysis of DHA oil was carried out with a second addition of 5% enzyme loading and was monitored for a longer period of time, in order to reach a similar conversion to that of ARA oil (Figure 1B). As can be observed, 72 h was necessary for a ca. 90% FFA level in ARA oil and 96 h for DHA oil. Other authors have also observed a faster hydrolysis rate for FAs with a chain length shorter than C22 compared to that attained for DHA [15].

### 2.2. Production of Predigested Mixtures

**Figure 2:**
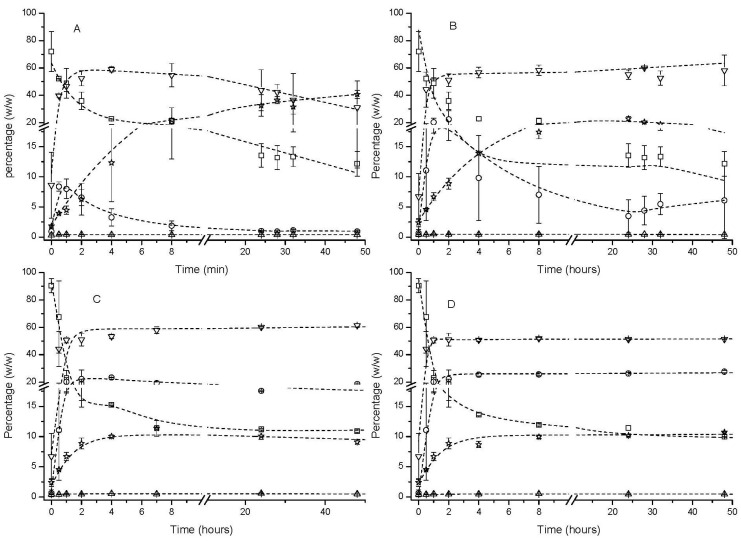
Time course of esterification reaction between mixtures of fatty acids from ARA oil and DHA oil in a ratio of 2 to 1 (*w*/*w*) and glycerol. 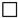 FFA: free fatty acid, 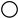 MAG: monoacylglyceride, 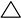 Cholesterol, 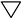 DAG: diacylglyceride, 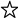 TAG: triacylglyceride. Esterification at 55 °C and at FFA/glycerol ratio 1:1 (**A**) and 1:3 (**B**). Esterification at 20 °C and at FFA/glycerol 1:3 (**C**). Esterification at 10 °C and at FFA/glycerol 1:3 (**D**).

Different reaction conditions were studied for the esterification reaction between the mixtures of fatty acids from ARA oil, DHA oil, and glycerol. First, the reaction was carried out at 55 °C at two molar ratios of FFA and glycerol, namely 1:1 and 1:3 (Figure 2A,B). At stoichiometric conditions, only 8% of monoacylglycerols (MAGs) are formed after 1 h of esterification. Then, MAGs start decreasing. At these conditions, more than 40% of triacylglycerols (TAGs) are formed after 48 h. As the objective of this esterification reaction is the production of an acylglycerol mixture rich in MAGs, stoichiometric conditions were discarded. At 55 °C and utilizing a three times molar excess of glycerol (Figure 2B), the amount of MAG increased up to 18% after 1 h of the esterification reaction. However, after that, MAGs started decreasing, reaching values of 5% after 48 h of the reaction. At these conditions, only ca. 20% of TAGs are produced after 48 h of the reaction. In order to preserve the percentage of MAGs as high as possible, the temperature was decreased to 20 °C utilizing a molar ratio of FFA to glycerol of 1 to 3 (Figure 2C). It can be observed that at 20 °C, the amount of MAG was ca. 20% after 2 h of the reaction and then started decreasing slowly up to 18% after 48 h. In addition, only 10% of TAGs was observed after 48 h of the reaction. To increase the content of MAGs even more, an esterification reaction utilizing a molar ratio of FFA to glycerol of 1 to 3 was also carried out at 10 °C (Figure 2D).

It has been previously reported that the formation of MAGs in an enzymatic esterification reaction is increased at lower reaction temperatures [16]. This fact has been attributed to MAG partial solidification at low temperatures that separate it from the reaction mixture. At these reaction conditions, MAGs are not completely liquid in the reaction mixture and suffer very low esterification to produce diacylglycerols (DAGs). This way, they are kept almost constant along the esterification reaction. For this reason, MAGs reached a maximum content of ca. 25% *w*/*w* that was kept constant along 48 h of the reaction. Similarly to the reaction at 20 °C, TAGs never exceeded 10% of the reaction mixture. This mixture also contained ca. 40% of DAGs. It should be remarked that the first two hours of the reactions at 20 and 10 °C were performed at 55 °C to maximize MAG production and maintain it thereafter without the need for organic solvents. Other authors have achieved up to 68% MAGs after 72 h in an esterification reaction with an FFA excess (6:1, FFA/glycerol), in the presence of organic solvent and dissolved lipase [17]. Yang et al. [18] have carried out an esterification with Novozym 435 in a solvent-free medium with the aim of obtaining a reconstituted TAG and reducing the FFA content. Meanwhile, we want to obtain a product rich in MAGs, DAGs, and FAs to simulate a predigested product (Figure 2D).

### 2.3. In Vitro Digestion

**Figure 3:**
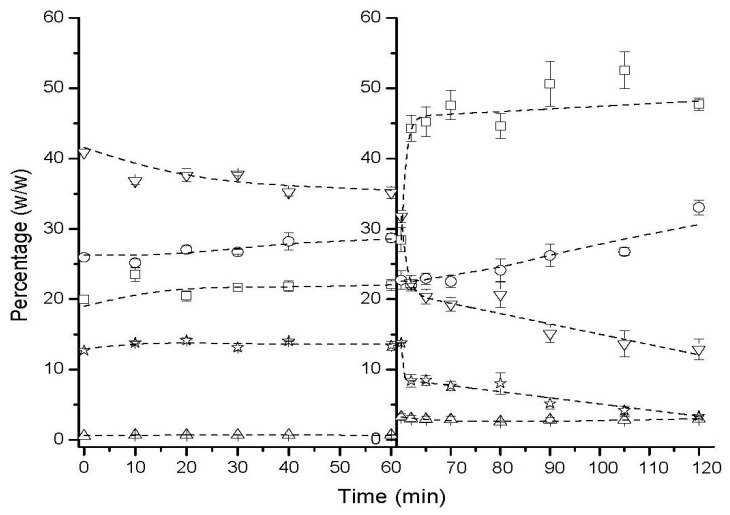
Time course of in vitro gastric and intestinal digestion of predigested product. 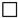 FFA: free fatty acid, 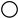 MAG: monoacylglyceride, 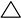 Cholesterol, 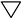 DAG: diacylglyceride, 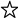 TAG: triacylglyceride.

The esterification product under optimal conditions (Figure 2D) was subjected to an in vitro digestion process. As can be observed in Figure 3, the hydrolysis, in the gastric phase (first 60 min), of the predigested product (esterification product obtained at 10 °C) is very low. DAGs decreased around 5% by weight, producing a slight increase in MAGs and FFAs. TAGs were almost constant along the gastric phase. Because gastric lipase prefers short- and medium-chain fatty acids and rejects long-chain fatty acids, in addition, gastric lipase has been shown to be active at pH levels ranging from 2 to 8 and continues to hydrolyze TAGs during the intestinal phase [19,20]. When intestinal digestion begins, the first stage of a rapid hydrolysis rate was observed (corresponding to the first 5 min of the intestinal stage) and a slower hydrolysis rate in the rest of the intestinal stage. Approximately 50% of fatty acids are released at the end of the in vitro digestion, and ca. 30% of MAGs are attained at the end of this stage. It is worth mentioning that other studies in which DHA-rich microalgae oil emulsions or infant formulas have been digested only determine the NEFA content during digestion and unfortunately do not assess the evolution of other lipid classes [20,21].

#### 2.3.1. Digestion Product

The digestion product (DP) obtained after the intestinal phase was extracted and analyzed by gas chromatography. This product comprised ca. 52% free fatty acids, 30% MAGs, 3% cholesterol, 12% DAGs, and 3% TAGs. A very small amount of undigested TAGs and DAGs and a very high content of MAGs are observed that could be indicative of an adequate digestibility of this product, confirming the good digestibility of the esterification product since it has been shown that a digested lipid with less than 10% of MAGs in the final digestion products can be considered of poor digestibility and consequently low bio-accessibility [3,22]. Unfortunately, numerous papers focused on in vitro digestion, only describing the hydrolysis percentage to explain digestibility [21,23]. All these publications do not pay attention to the acylglycerol composition of the DP, and they do not provide adequate information regarding the DP. As an example, if MAGs are readily digested as they are formed, because they are more easily digested than DAGs or TAGs, an evaluation of the hydrolysis level is not an adequate parameter to characterize this phenomenon. For this reason, monitoring all different acylglycerols besides the FFAs released should be carried out to properly describe in vitro digestion. Moreover, the hydrolysis level does not allow for the determination of the mass balance of the process after phase separation, because other chemical species contained in the DP are not considered. Additionally, digestibility should be based on the FFA and MAG level and not only on the FFAs released. 

**Figure 4:**
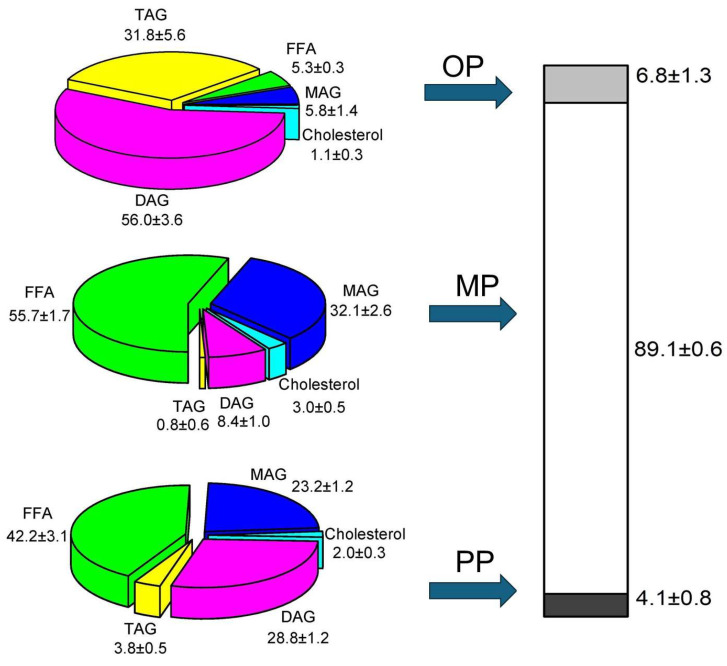
Phase distribution and composition after in vitro digestion of predigested product. 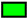 FFA: free fatty acid, 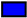 MAG: monoacylglyceride, 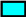 Cholesterol, 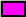 DAG: diacylglyceride, 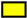 TAG: triacylglyceride. 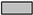 OP: oil phase, 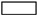 MP: micellar phase, 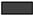 PP: precipitate phase.

The DP was also centrifuged, and three phases were obtained (Figure 4), namely the oily phase (OP), micellar phase (MP), and precipitate phase (PP). Approximately 90% (*w*/*w*) of the digestion product was found in the MP, which is considered the bio-accessible phase. Remarkably, less than 7% of the DP was OP, which indicates a very small nondigestible fraction. Finally, a very small PP (4%), which contains insoluble lipid materials from the digestion product at 37 °C, was also attained. Regarding the composition of the OP, it was formed mainly by TAGs and DAGs that were not digested. The MP consisted mainly of free fatty acids and MAGs, and the PP comprised glycerides and FFAs that are solid at 37 °C. Taking into account all this information, it can be stated that this predigested mixture it has very adequate in vitro digestibility, since the presence of an emulsifier in the medium favors lipid digestion [24], favoring their dispersion at the onset of digestion, which may enhance both the hydrolysis of TAGs and DAGs and the creation of micellar structures, allowing the DP to be more easily absorbed. Again, this has also been noted in previous work on the in vitro digestion of the GP from shark liver oil [25].

#### 2.3.2. The Distribution of Each Lipid Compound among the Different Phases

**Figure 5:**
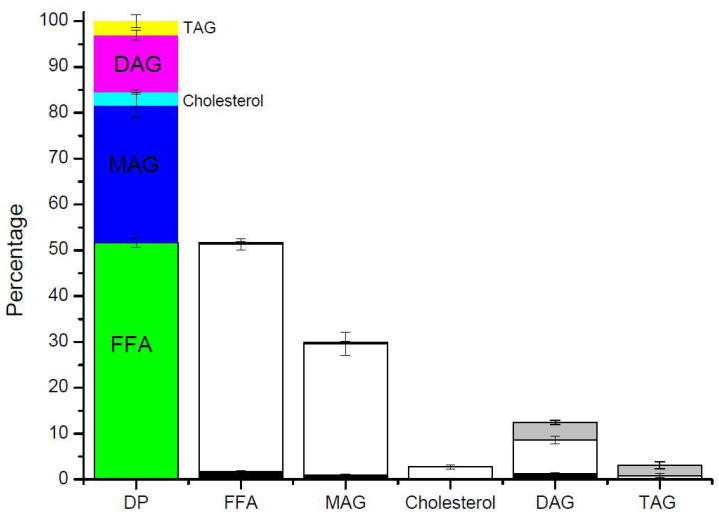
The distribution of each lipid compound among the different phases. 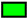 FFA: free fatty acid, 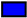 MAG: monoacylglyceride, 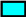 Cholesterol, 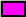 DAG: diacylglyceride, 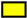 TAG: triacylglyceride. 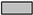 OP: oil phase, 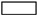 MP: micellar phase, 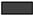 PP: precipitate phase.

The composition of each of the phases determined by GC was used to find out how each lipid class is distributed between the OP, MP, and PP. When examining the percentage of each lipid class in each phase obtained after centrifugation (sector diagrams in Figure 4), it is not possible to know the relative amount or content of each lipid in each phase. It is important to know how each lipid class distributes in each phase to know which lipid compounds are more bio-accessible (Figure 5). To properly understand this, it is important to correlate the percentage of each species in the digestion product, the proportion of each phase obtained after centrifugation, and the percentage of each lipid class in each phase (sector diagrams in Figure 4). The OP contains around 5% of FFAs, which represents 6.8% of the total digestion product. Therefore, 0.4 parts out of 51.7 parts of FFAs in the digestion product are present in the OP. If we express this value in a percentage, it corresponds to ca. 0.7% of the total FFAs in the OP. Furthermore, by adding this relative content of each lipid in each phase, the proportion of each phase obtained after centrifugation is obtained.

If we want to know the distribution of MAGs among the three phases, we proceed in a similar way. Therefore, if we have 30% of MAGs in the DP and 0.4 parts of these 30 parts are in the OP, 1.3% of the total MAGs are in the OP. It can be concluded that FFAs and MAGs are found mainly in the MP (96 and 95.5%, respectively), and TAGs are located mainly in the OP (72%, respectively). In this case, surprisingly, 60% of the total DAGs are located in the MP, which could indicate that the MP has excellent emulsifying properties, and it is able to incorporate a significant portion of DAGs.

## 3. Materials and Methods

### 3.1. Materials

Arachidonic fatty acid-rich oil (ARA oil) was supplied by Penta Manufacturing Company (West Caldwell, NJ, USA) and stored in a modified atmosphere of nitrogen at −20 °C. Docosahexaenoic fatty acid-rich oil (DHA oil) sourced from the marine microalgae *Schizochytrium* sp. was supplied by Progress Biotech (Capelle aan den Ijssel, The Netherlands) and stored under a modified atmosphere of nitrogen at −20 °C. The lipid composition and the initial oxidative status of ARA and DHA oils are shown in Table 2. It can be observed that most of the lipid species are TAGs, present in 89.82% and 95.09%, respectively. Both ARA oil and DHA oil possess adequate oxidation status, as both values are within the established limits for human consumption [26]. The main fatty acids present in the ARA-rich oil were determined using GC to be arachidonic acid 53.8%, stearic acid 8.3%, oleic acid 8.3%, palmitic acid 7.1%, linoleic acid 6.2%, and γ-linolenic acid 4.9%. Regarding microalgae oil, the main fatty acids were DHA 54.8%, palmitic acid 17.6%, docosapentaenoic acid 10%, and oleic acid 9.4%.

Reagents used for in vitro digestion, trizma, pepsin, maleic acid, bile salts (BSs), and cholesterol, were purchased from Sigma-Aldrich (St. Louis, MO, USA). Phosphatidylcholine from egg yolk (PC) was supplied by Lipoid (Ludwigshafen, Germany); food-grade phospholipase A2 (PLA2) from Streptomyces violaceoruber (103 U mg^−1^) was supplied by Nagase Chemtex Corporation, Fukuchiyama Factory (Kyoto, Japan). Pancreatin from porcine pancreas was purchased from MP Biomedicals, LLC (Irvine, CA, USA). Hydrochloric acid, sodium chloride, and calcium chloride were from Panreac (Barcelona, Spain). Rabbit gastric lipase was supplied by Lipolytech (Marseille, France) and stored in freezer storage. Hexane (HEX), methyl-tertbutyl ether (MTBE), petroleum ether (PE), chloroform, and methanol were supplied from Macron (Avantor Performance Materiale, Center Valley, PA, USA) and formic acid (98% purity) from Panreac (Barcelona, Spain). All these solvents were of HPLC and GC grade. Pure standards of ARA oil and DHA oil (MAG and DAG) were obtained by Solid-Phase Extraction (SPE) using silica gel from Supelco–Merck Group (Darmstadt, Germany). The step-elution was carried out according to the method described by Ingalls [27]. For this purpose, the glycerolysis of ARA oil and DHA oil in a 2 to 1 (*w*/*w*) ratio was carried out according to the method described by Corzo et al., for which the Novozym^®^ 435 enzyme (Novozymes, Bagsværd, Denmark) and glycerol (Scharlab S.L., Mas d’En Cisa, Spain) were used. A liquid lipase from the genetically modified *Aspergillus oryzae* microorganism (commercially known as Eversa Transform^®^ 2.0) and Novozym 435 lipase were kindly donated by Novozymes (Bagsvaerd, Denmark).

### 3.2. Methods

#### 3.2.1. Enzymatic Hydrolysis of Microalgae and Arachidonic Acid Oils

The enzymatic hydrolysis of microalgae (DHA oil) and arachidonic acid (ARA oil) oils catalyzed by Eversa^®^ Transform 2.0 was carried out at 45 °C in a 250 mL reactor with a mechanical stirrer at 300 rpm. A total of 25 g of oil, 25 g of distilled water, and 10% (*w*/*w*) of enzyme loading with respect to the amount of oil added were mixed. Furthermore, the hydrolysis reaction of microalgae oil was supplemented with an additional 5% (*w*/*w*) of enzyme loading after 32 h of the reaction. Aliquots of 0.5 mL were taken at the following reaction times: 2 h, 4 h, 8 h, 24 h, 32 h, 48 h, 56 h, 72 h, and 96 h, which were then deposited in a 2 mL Eppendorf. Subsequently, these samples were centrifuged at 12,000 rpm for 2 min in a ScanSpeed Mini microcentrifuge (Skanderborg, Denmark). Three phases were separated: an upper oily phase, an interface where lipase is located, and a lower aqueous phase consisting mainly of water, glycerin, and other polar residues. The oily (lipid) fraction was analyzed to determine the FFA content. 

This lipid fraction was utilized to determine the acid value, according to the official method AOCS Ca5a-40, with slight modifications (AOCS, 2009). A total of 50 mg of the sample was weighted in a flask and dissolved with 25 mL of ethanol/diethyl ether 1:1. Then, 5–6 drops of a 1% methanolic solution of phenolphthalein (95% *v*/*v*) was used as an indicator. These mixtures were titrated with KOH 0.1 N. KOH that was previously standardized with potassium phthalate to determine the exact concentration according to the official method AOCS H 12-52 (AOCS, 2009). 

The acid value was determined according to the following formula:

(1) $$\mathrm{acid}\;\mathrm{value}\;(\%)=\frac{V\;\times \;M\;\times \;N}{10\;\times \;W}$$

V: mL of KOH; N: KOH Normality; M: the molecular weight of the free fatty acid; and W: sample weight.

The final hydrolysis products were stored in light-protected flasks under nitrogen atmosphere and at 4 °C, until their further use.

#### 3.2.2. Enzymatic Esterification

The enzymatic esterification of a mixture comprising FFAs from ARA oil and FFAs from microalgae oil (DHA oil) mixed in a 2:1 ratio (*w*/*w*) was carried out in the presence of Novozym 435 lipase. Different temperatures and FFA/glycerol ratios were studied. In each reaction, 6.66 g of FFAs from hydrolyzed ARA oil, 3.33 g of FFAs from hydrolyzed DHA oil, and 10% (*w*/*w*) of the lipase were mixed. The percentages of glycerin studied were as follows: 10% (*w*/*w*) and 30% (*w*/*w*) of total FFAs. Three different temperatures were investigated, namely 10, 22, and 55 °C. The reactions were carried out with magnetic stirring. The reactions at 22 °C and 10 °C were first carried out for 2 h at 55 °C; then the reaction mixture was introduced into an IKA KS 4000 orbital incubator at 300 rpm. Aliquots of 0.4 mL were taken at the following reaction times: 0 h, 1 h, 2 h, 4 h, 8 h, 24 h, 32 h, and 48 h. Additional aliquots at 72 and 96 h were taken in the reactions at 22 °C and 10 °C. All reactions carried out were carried out in duplicate. The aliquots were placed in 2 mL capacity Eppendorfs, and 1 mL of chloroform was added. Subsequently, it was filtered with a 25 mm and 0.45 μm hydrophilic polyvinylidene difluoride (PVDF) membrane filter (Symta, Madrid, Spain).

Finally, each sample was taken to an evaporator under nitrogen purge (Stuart Block Heater SBH200D/3, Fisher Scientific, New Hampton, NY, USA) until a constant weight of oily residue was obtained. From each oily residue obtained, the FFA content was determined by means of the acid value previously described. In addition, the MAG, DAG, COL, and TAG content was determined via GC analysis.

#### 3.2.3. Lipid Identification and Quantification by Gas Chromatography

GC analyses were carried out in a Gas Chromatograph Agilent 7820A (Agilent Technologies, Santa Clara, CA, USA) with on-column injection coupled to an Flame Ionization Detector (FID), according to the method described by Torres [28]. The used column was an HP-5MS capillary column, 5% phenyl methyl silicone (length 7 m, internal diameter 0.25 mm, and thickness 0.25 µm). The injection volume was 0.1 µL. The temperature of the injector and detector was 50 and 340 °C, respectively. The program of temperatures started at 60 °C, increasing at 42 °C min^−1^ until 250 °C. This temperature was maintained for 20 min and then increased up to 340 °C at 25 °C min^−1^, which was maintained for 35 min. The quantification was performed by the external standard method using pure standards. For this, calibration curves were made with each of the standards. The data are expressed as the percentage of TAGs, DAGs, MAGs, and FFAs with respect to the weight of the residue (w.r.) obtained after lipid extraction, according to the following equation:

(2) WcompWw.r.×100

where W_comp_ is the weight of each type of lipid, and W_w.r._ is the weight of the residue obtained after lipid extraction.

The distribution of each lipid type was determined by combining the relative weight of each phase and composition according to the following equation:

% of A = weight percent of oil phase × (percentage of A in oil phase/100) + weight percentage of the micellar phase × (percentage of A in the micellar phase/100) + weight percentage of the precipitated phase × (percentage of A in the precipitated phase/100)(3)

where A refers to any of the types of lipids analyzed in this study.

#### 3.2.4. In Vitro Gastrointestinal Digestion Model

An in vitro gastrointestinal digestion model was carried out in two stages, a first stage of gastric digestion and a second stage of intestinal digestion, based on the study from Chabni et al. [3].

##### Gastric Digestion

Fat samples of the esterification product obtained at 10 °C, which will be called the predigested product from now on (1.86 g), were pre-emulsified with 7.44 mL of gastric phase simulation fluid (SFG) in a digester jacketed with temperature control at 37 °C and constant stirring at 900 rpm for 10 min, using a mechanical stirrer (Metrohm, Herisau, Switzerland), after sonication with an ultrasound probe (Vibra cell, VC 130 (Sonics, Newtown, CT, USA)) for 1 min at 70%. Gastric digestion began by adding 1.16 mL of a solution previously prepared, consisting of 400 mg of rabbit gastric lipase (activity > 15 U mg^−1^ of protein) and 24 mg of porcine pepsin (EC 3.4.23.1) in 2.5 mL of distilled water, which was kept under stirring at 900 rpm for 10 min. Gastric digestion was carried out for 60 min at 37 °C and 900 rpm.

In order to study the evolution of lipid products during the hydrolytic process that takes place in gastric digestion, aliquots of 400 μL were taken at the minutes 10, 20, 30, 40, and 60. At minute 60, a sample of 2.925 mL was also taken, with the goal of starting with 1 g of fat in intestinal digestion.

##### Intestinal Digestion

After gastric digestion, 26 mL of Trizma-maleate buffer (0.1 M, pH 7.5) was added to the stirred mixture. To simulate biliary secretion, a solution was prepared consisting of 500 mg of bile salts, 200 mg of egg yolk phosphatidylcholine, 40 mg of cholesterol (Sigma-Aldrich Chemie GmbH, Steinheim, Germany), 1 mL of a 325 mM CaCl_2_ solution, 3 mL of a 3.25 mM NaCl solution (Panreac Química S.A.U, Barcelona, Spain), and 20 mL of 0.1 M Trizma-maleate buffer, pH 7.5. This mixture was homogenized with ultra-turrax T18 basic IKA (Staufen, Germany) for 2 min at 3500 rpm. Afterwards, the simulation bile secretion was added to the resulting emulsion after gastric digestion, and all of it was homogenized again with ultra-turrax for 2 more minutes at 3500 rpm. Intestinal digestion began by adding a solution of fresh pig pancreatin extract prepared from 100 mg of pancreatin in 4 mL of Trizma-maleate buffer 0.1 M pH 7.5, stirred at 900 rpm for 10 min and centrifuged at 1600 G, at 5 °C for 15 min. The supernatant was added to the reaction medium together with 2.5 mL of phospholipase A2 food grade. The intestinal phase was carried out for 60 min at 37 °C and 900 rpm.

In order to study the evolution of lipid products during the hydrolytic process that takes place in intestinal digestion, aliquots of 1 mL were taken at the minutes 2, 5, 10, 20, 30, 45, and 60 being the final digestion product (DP). In addition, an aliquot was also taken at minute 0 of the intestinal digestion, i.e., before adding the pancreatin solution.

##### Phase Separation after In Vitro Digestion

After 60 min of intestinal digestion, the digestion products were centrifuged at 4500 rpm and 37 °C for 45 min (Sorvall 6000 LYNX, Thermo Fisher Scientific, Waltham, MA, USA) according to the method described by Martin et al. [29]. After centrifugation, three phases were obtained: an upper oily phase (OP), formed by the undigested lipid fraction; an intermediate aqueous fraction, called the micellar phase (MP), which contains the lipid fraction digested in the form of micellar and vesicular structures; and a precipitate phase (PP), which consists of a lower phase containing insoluble compounds at 37 °C. These three phases were separated from each other for the subsequent extraction of the lipids present in each of them.

##### Lipid Extraction

The total lipids contained in the three phases obtained (OP, MP, and PP) were extracted from the samples in three sequential steps using solvent mixtures with increasing polarity in a solvent/sample ratio of 3:1 (*v*/*v*) and centrifuging for 10 min at 14,500 rpm each time [25], with the exception of the PP, which was extracted with a solvent/sample ratio of 6:1 (*v*/*v*). The solvents used were n-hexane/methyl-tert-butyl-ether (MTBE) (50:50, *v*:*v*); MTBE/Petroleum ether (PE) (50:50, *v*:*v*); and PE/ethanol (1:0.6, *v*:*v*). After each extraction, the supernatant was collected in an empty vial, and possible impurities were allowed to decant. Later, the supernatant was transferred to another previously weighed vial and evaporated under a stream of nitrogen using a Stuart Block Heater SBH200D/3 (Staffordshire, UK) to a constant weight of the residue (w.r.). Finally, the samples were diluted with acidified chloroform (0.1% formic acid) to a final concentration of 6 mg mL^−1^ before injection in the GC system according to Section 3.2.3. 

### 3.3. Statistical Analysis

Data analysis was performed using Excel 2010 (Microsoft Office), and all statistical evaluations were performed using Origin (version 9.0 for Windows; OriginLab Corporation, Northampton Northampton, MA, USA). Experiments were carried out in duplicate, and the data were expressed as the mean ± standard deviation. The statistical significance of the differences between the groups was measured by a one-way analysis of variance (ANOVA) and post hoc Tukey HSD test. Statistical significance was defined at the level of *p* < 0.05.

## 4. Conclusions

The predigested product described in this work has excellent bio-accessibility, considering the very low percentage of undigested TAGs in the digestion product (3%), the abundant content of FFAs (50%) and MAGs (30%), and the percentage of the MP after centrifugation (90%). All these data are indicators of a very adequate digestibility. 

As the main drawback of the predigested mixture utilized in this study, the production of this mixture requires two enzymatic steps (hydrolysis and esterification) and the utilization of low temperatures (10 °C) that can increase the market price of the product and reduce their industrial feasibility. Moreover, the initial content of FFAs (ca. 20%) of this product could also increase the oxidative rancidity and reduce its half-life.

Additional oxidation stability studies and in vivo tests of bioavailability are needed to corroborate the exceptional bio-accessibility of the product developed in this study.

## Figures and Tables

**Table 1:** Rate of hydrolysis of ARA oil and DHA oil.

	Slope	Standard Error	Adj. R-Square
DHA oil	−0.04435	0.0032	0.98458
ARA oil	−0.10494	0.01697	0.9254

**Table 2:** Lipid composition (%) and oxidative status of arachidonic fatty acid-rich oil (ARA oil) and docosahexaenoic fatty acid-rich oil (DHA oil). Values are means ± SD.

Initial Oxidative Status	ARA Oil	DHA Oil
Peroxide value	3.45 ± 0.05	5.39 ± 0.74
p-anisidine value	5.65 ± 0.15	1.90 ± 0.42
TOTOX	12.55 ± 0.05	12.67 ± 1.91
**Lipid profile (g/100 g)**		
Triglycerides (TAGs)	89.82 ± 0.08	95.09 ± 0.03
Diglycerides (DAGs)	4.30 ± 0.05	4.27 ± 0.04
Monoglycerides (MAGs)	0.33 ± 0.08	0.26 ± 1.85
Free fatty acids (FFAs)	4.68 ± 0.03	0.24 ± 0.04
Cholesterol (CHOL)	0.87 ± 0.04	-

## Data Availability

The data presented in this study are available in the article, further inquiries can be directed to the corresponding author.
